# Multimedia Informed Consent Tool for a Low Literacy African Research Population: Development and Pilot-Testing

**DOI:** 10.4172/2155-9627.1000178

**Published:** 2014-04-05

**Authors:** Muhammed Olanrewaju Afolabi, Kalifa Bojang, Umberto D’Alessandro, Egeruan Babatunde Imoukhuede, Raffaella M Ravinetto, Heidi Jane Larson, Nuala McGrath, Daniel Chandramohan

**Affiliations:** 1 Medical Research Council Unit, Atlantic Road, Fajara, the Gambia and London School of Hygiene and Tropical Medicine, Keppel Street, WC1E 7HT, UK; 2 London School of Hygiene and Tropical Medicine, Keppel Street, WC1E 7HT, UK; 3 Centre for Clinical Vaccinology and Tropical Medicine, the Jenner Institute, Oxford University Churchill Hospital, Oxford, OX3 7LJ, UK; 4 Institute of Tropical Medicine, Antwerp & KU Leuven, Belgium; 5 University of Southampton, Southampton, Mailpoint 805, C floor, South Academic Block, Southampton General Hospital, Southampton, SO16 6YD, UK

**Keywords:** Informed consent, Video, Technology, Vulnerable population, Development, The Gambia

## Abstract

**Background:**

International guidelines recommend the use of appropriate informed consent procedures in low literacy research settings because written information is not known to guarantee comprehension of study information.

**Objectives:**

This study developed and evaluated a multimedia informed consent tool for people with low literacy in an area where a malaria treatment trial was being planned in The Gambia.

**Methods:**

We developed the informed consent document of the malaria treatment trial into a multimedia tool integrating video, animations and audio narrations in three major Gambian languages. Acceptability and ease of use of the multimedia tool were assessed using quantitative and qualitative methods. In two separate visits, the participants’ comprehension of the study information was measured by using a validated digitised audio questionnaire.

**Results:**

The majority of participants (70%) reported that the multimedia tool was clear and easy to understand. Participants had high scores on the domains of adverse events/risk, voluntary participation, study procedures while lowest scores were recorded on the question items on randomisation. The differences in mean scores for participants’ ‘recall’ and ‘understanding’ between first and second visits were statistically significant (F (1,41)=25.38, p<0.00001 and (F (1, 41) = 31.61, p<0.00001 respectively.

**Conclusions:**

Our locally developed multimedia tool was acceptable and easy to administer among low literacy participants in The Gambia. It also proved to be effective in delivering and sustaining comprehension of study information across a diverse group of participants. Additional research is needed to compare the tool to the traditional consent interview, both in The Gambia and in other sub-Saharan settings.

## Introduction

Informed consent is a practical application of respect for person which is a fundamental principle of research [1]. The process of informed consent is meant to ensure that the decision to participate in clinical research is freely made after an individual has received, considered and understood fully the complete study information without being coerced, induced, unduly influenced or intimidated [2]. For informed consent to fulfil its goals researchers must not only provide full disclosure of study information but they must also ensure full comprehension of the information to potential study participants. Informed consent must be provided in the languages and terminologies understandable to the participants to allow him/her understand the study information to make an informed decision on whether or not to participate [2,3].

Comprehensive systematic reviews [4,5] of clinical research conducted in sub-Sahara Africa showed that participants often demonstrated poor comprehension of various domains of informed consent. For example, only 10% of mothers of study children in The Gambia [6] and 20% of Ghanaian women [7] demonstrated good comprehension of the concept of placebo. Similarly, participants from Ivory Coast [8], Nigeria [9], Senegal [10], Kenya [11], Uganda [12] and Ethiopia [13] had sub-optimal comprehension of voluntary participation, autonomy, risks/benefits, randomisation and blinding. These unacceptably low levels of comprehension may be due to a combination of factors. These include poor communication between the participants and the persons administering the consent (e.g. due lack of time and power imbalance making the participants not to ask necessary questions).

Furthermore, central to this problem is the almost exclusive reliance on written information document in research settings where many study participants are unable to read and understand documents written either in foreign or local languages [5,14,15]. In such situations, the World Medical Association Declaration of Helsinki recommends the use of appropriate alternative informed consent procedures that will engender adequate comprehension of the study information [16,17].

Nishimura et al. [18] in a recently published systematic review identified several effective strategies for improving informed consent process including enhanced consent forms, extended discussions and multimedia. Enhanced consent form involved the use of simplified paper consent document with revised layout, text styling, and sometimes with added pictures. In extended discussion, a study team member engaged participants in additional discussions and, multimedia approach involved presentation of study information through combined use of video, audio and animations [19]. A meta-analysis showed a modest but statistically non-significant increase in comprehension scores of participants randomised to a multimedia-based consent approach when compared to their counterparts randomised to control consent approach (Standardised Mean Difference [SMD]=0.30, 95% CI, −0.23 to 0.84). Similar comparison of enhanced consent form and extended discussion with control consent increased the comprehension scores significantly (SMD 1.73, 95% CI, 0.99 to 2.47; SMD 0.53, 95% CI, 0.21 to 0.84 respectively [18]. These findings raise concerns about the impact of multimedia in informed consent process, although, previous studies reported its usefulness in promoting retention of consent information longer than one week [20,21].

An earlier review by Flory and Emanuel [19] also showed that multimedia tools are not significantly effective in aiding participants’ comprehension. This submission agreed with the conclusions of a Cochrane review [22] that ‘the empirical literature is not yet sufficiently developed to draw definitive conclusions about the general effectiveness of or value derived from multimedia consent tools’**.** Nevertheless, critics argue that the reported limited usefulness of multimedia tool is untenable because the effectiveness of multimedia consent approach was evaluated in studies with considerable methodological flaws. For example, some of the studies had no standard controls, and in others, informed consent documents were merely presented on computer screens for participants to read [18]. Apart from these, the studies included in the systematic reviews [18,19,22] were conducted in developed countries where literacy rates were high. Consequently, the conclusions of lack of effectiveness of multimedia approach may not be applicable to low literacy populations in sub-Saharan Africa. Although no study from sub-Saharan Africa is yet to report the potential usefulness of multimedia for delivering study information during informed consent process; media-based technology is becoming cheaper to implement, readily available and more manageable in Africa [18,23]. Thus, assessing the effectiveness of a multimedia informed consent tool for improving the consenting process is becoming increasingly crucial in low literacy research settings in Africa.

As a first step, we describe here how we developed and pilot-tested a multimedia tool to provide information for a clinical trial scheduled to take place among low and non-literate participants in The Gambia. This study was also aimed at gathering preliminary data to determine the effectiveness of a locally designed multimedia tool in aiding informed consent comprehension of participants in the low literacy research community.

## Materials and Methods

### The PRINOGAM Trial

The development of the multimedia tool was done using the informed consent document for PRINOGAM trial (ClinicalTrials. gov NCT01838902). Briefly, PRINOGAM is an open-label, four-arm treatment trial, aimed at determining the lowest possible primaquine dose to obtain a substantial gametocytocidal effect in asymptomatic malaria infected individuals, as this may reduce the risk of harmful effects in Glucose-6-Phosphate Dehydrogenase (G6PD) deficiency.

The trial was planned to take place concurrently at Basse and Jahaly areas of The Gambia where level of literacy of the inhabitants is low. The Gambia is one of the smallest West African countries with a population of 1.79 million people and adult literacy rate of less than 30% [24]. Mandinka, Fula and Wolof are three major ethno-linguistically distinct groups populating the study areas. In previous studies [6,25] conducted in Gambian communities, the local ethic committees recommended that oral interpretations of the English version of written informed consent documents should be provided to potential participants by trained field staff who are native speakers of the local languages [26]. This is because, in addition to low literacy rate, no written translation of consent documents to local languages is possible as no standardised written format for the local languages exist [25,27]. Because most participants are not literate, they gave consent by thumb-printing the consent form in the presence of an impartial witness.

This pilot study of the multimedia consent tool was conducted among healthy volunteers in Basse, an area which shares similar epidemiologic and demographic features with Jahaly.

### Development of multimedia tool from informed consent document of PRINOGAM

We worked with a multimedia expert who had extensive training and experience in motion graphics and interactive media design to develop the participant information document of PRINOGAM trial. The information document was earlier written by the Principal Investigator of the trial with technical support from the Medical Research Council (MRC) Clinical Trials Support Manager who ensured that all relevant information was adequately and comprehensibly presented in the document. The document were submitted along with the study protocol to an independent body of scientists who reviewed and confirmed that information contained in the document was satisfactory to engender informed decision-making by potential study participants. The information document was further submitted to the local ethical committee who also reviewed and approved the document as conforming to internationally agreed ethical requirements for conduct of clinical trials.

The approved information document contained 11 sections namely: *introduction, reason for the study, what is G6PD, how to take part, what would happen if one took part in the study, what blood tests would be done, what are the side effects and possible risks of taking part, potential benefits, would taking part in this study be kept confidential, who has reviewed this study, who can be contacted if one has questions?* The messages in each section were graphically translated into a context-specific visual story. We serially reviewed these storyboards to confirm appropriateness to Gambia research setting. The stories were acted in role-plays by members of the clinical trial team after undergoing several training and rehearsals. The final role-play on each section of information sheet was serially video-recorded by the multimedia expert.

Three experienced linguistic professionals who are native speakers of the three major Gambian languages and are also familiar with clinical research concepts were contracted to audio-translate each section of the participant information document. The audio-translations were confirmed to be consistent with the English version by another three native speakers of the languages. The audio-translations were recorded as voice-over on the video-recorded role-plays by the multimedia expert. Sections which could not be visually conveyed in the role-plays e.g symptoms of adverse events of study drugs like headache, diarrhoea, passage of dark-coloured urine were graphically represented with animations.

### Review of multimedia tool

The first draft of the multimedia tool in a Digital Video Disc (DVD) was given to two qualified lay persons and two experienced researchers to confirm whether the contents of the tool was consistent with the contents of the participant information document of PRINOGAM trial. They all agreed that the tool included all essential information on the study as requested by ethical and Good Clinical Practice (GCP) guidelines, and that it used dialects that were well understandable to general populace. In very few areas, one of the narrators wrongly used a local language *‘biir bumuti’* which means ‘lower abdominal pain’ to describe ‘abdominal pain’ as one of the adverse effects of the investigational products. This was corrected with appropriate word ‘*nahl bumuti’.* Also, omission of ‘*hel butey’* meaning ‘nausea’ was discovered and this was included in the revised version. Non-inclusion of dark coloured urine as a major complication of G6PD deficiency was pointed out by one of the researchers and this was included in the revised version.

### Pilot-testing

A purposive sample of 42 healthy male and female volunteers aged 18-49 years was recruited to pilot-test the multimedia informed consent tool. The upper limit of the age range (49 years) was based on data from previous studies [28,29]. The lower age limit (18 years) was chosen to avoid the logistical challenges associated with obtaining informed consent from under-aged participants. Participants were recruited from the north and south parts of Basse to ensure representation. Despite being representative of the PRINOGAM trial population and participants could in the future become eligible, they were not screened for PRINOGAM when the pilot-testing was carried out. After obtaining a written informed consent, we played the multimedia tool on a laptop computer for each participant in his/her preferred local language in noise-free consulting rooms at MRC facilities located within Basse Major Health Centre. The participants were requested to ask questions if they were not clear about the contents of the multimedia tool.

To assess acceptability and ease of use of the multimedia tool, an 8-item questionnaire was adapted from a similar study conducted in South Africa [30]. The original questionnaire contained 15 question items on acceptability and ease of use of an alternative informed consent tool. The relevant part of the question items were retained e.g. “*do you like the pictures in the tool*” while non-relevant questions were removed e.g “*do you know how to replace the battery of the tool*”. After watching the multimedia video and participants confirmed they had no questions, the 8-item questionnaire was administered to each participant to assess acceptability and ease of use of multimedia tool. Participants responded by indicating either ‘yes’ or ‘no’ to each question item. Following the questionnaire administration, we assessed the participants’ comprehension using a Digitised Audio Informed Consent Comprehension Questionnaire (DICCQ) that was previously validated in low literacy Gambian populations. The development and psychometric evaluation of DICCQ has been described elsewhere [31]. Briefly, DICCQ is a 26-item questionnaire consisting of a combination of closed-ended, multiple choice and open-ended question items. Psychometric evaluation of DICCQ done in urban and rural Gambian populations showed that the questionnaire was reliable and valid [31]. The question items in DICCQ are consistent with the elements of informed consent required to ensure understanding of potential participants according to international ethical guidelines [2,32]. The following domains of informed consent are covered in the DICCQ: voluntary participation (Q1), rights of withdrawal (Q2,8,11,15), study procedures (Q17, 18,21,22), study purpose (Q16,20), blinding (Q3,4), confidentiality(Q5), compensation (Q9,14), randomization (Q10,23), autonomy (Q13), meaning of giving consent (Q12), benefits (Q19), risks/adverse effects (Q25), therapeutic misconception (Q24), placebo (Q26). The question items in DICCQ are listed in Table 4.

We further assessed acceptability and ease of use of DICCQ using the questionnaire adapted from the South African study described above [30].

To assess how much of the study information was retained, the participants were recalled one week after first administration and the digitised comprehension questionnaire was re-administered to the participants.

### Focus group discussions

During the second visit, selected participants were invited for Focus Group Discussions (FGD) to further explore acceptability and ease of use of multimedia consent tool and digitised informed consent comprehension questionnaire. A separate group of six men and women were invited for the FGD sessions. Participants were segregated by sex to ensure homogeneity and open discussions in each group. A purpose-designed FGD guide was used and the first author, MOA, served as the facilitator of the discussions. The proceedings were audio-taped after verbal consent was obtained from the participants. These were transcribed into English texts by two independent native speakers. We identified the main themes of the transcribed texts and content analysis of these themes was performed.

### Ethical consideration

Ethical approvals were obtained from the ethics committees of London School of Hygiene and Tropical Medicine, UK and Gambia Government/Medical Research Council Joint Ethics Committee. Due to absence of standardised writing formats for Gambian languages and high illiteracy rates, informed consent was obtained from the participants in this study by trained field assistants who were native speakers of the local languages. The trained assistants provided oral interpretations of the study information to the participants in the local languages he/she understood. After the potential participants had agreed to join the study, literate participants (about 10% in this study) signed the consent form while the majority (about 90%) thumb-printed the consent form in the presence of an impartial witness. Participation was voluntary and confidential.

#### Scoring system

The scoring system used in previous validation work of DICCQ [31] was applied:

**Table:** 

Closed ended question items in the first section	Each correct answer was scored 3; wrong answer was scored 0 and responses with ‘I don’t know’ were scored 1
Open-ended question items which are follow-up questions to the closed ended question items in the first section	Each correct answer was scored 5, partially correct answer was scored 3, incorrect answer was scored 0, while ‘I don’t know’ responses were scored 1
In the second section, participants chose ONE correct answer out of FOUR option responses	Each correct answer was scored 3, incorrect answer was scored 0 or ‘I don’t know’ responses were scored 1
In the third section, participants chose more than one correct answers from FOUR option responses	Full correct answers were scored 4, partially correct answers were scored 2, wrong answers were scored 0 and ‘I don’t know’ answers were scored 1
In the fourth section, participants responded using their own words to open-ended question items	Full correct answer was scored 5, partially correct answers were scored 3, wrong answers were scored 0 and ‘I don’t know’ responses were scored 1

### Data analysis

Data on acceptability and ease of use of multimedia tool and digitised questionnaire were entered on Microsoft Excel while data on participant comprehension were retrieved from the in-built database within DICCQ and exported into Microsoft Excel. Acceptability and ease of use were assessed by calculating the percentage of ‘yes’ responses indicated by participants on the questionnaire. Mean participants’ scores (and standard deviations) on DICCQ were calculated to determine the domains of informed consent which were most or least understood by the participants.

Further analysis was done by adopting the definition of comprehension used by Minnies et al. [33] which consists of two components: recall and understanding. ‘Recall’ was defined as correct answers to the close-ended and multiple choice questions while ‘understanding’ was correct responses given to the open-ended questions [33].

Repeated measures analysis of variance model was done to determine the effect of the multimedia and the effect of time on the participants’ recall and understanding scores at the two study visits. Pair-wise comparison of the mean difference of participants’ scores between first and second visits was performed and appropriate Bonferroni corrections were made to allow for multiple comparisons. Analysis was done with Stata version 12.1 (College Station, USA) with p<0.05 (two-tailed) considered significant.

## Results

Forty-two participants consisting of 20 females and 22 males were recruited. Table 1 shows socio-demographic characteristics of the study participants. The median age was 34.5 ± 11; (range, 18-48 years), 90% were Mandinka and less than 10% had Western education. Each playing session of the multimedia tool lasted an average of 20 minutes, while questionnaire administration through DICCQ took an average of 32 minutes.

All participants liked the features of the multimedia tool, would like to use it again, and wanted future study information delivered using the tool (Table 2). About 70% reported that they were comfortable with the tool and that it was easy to follow. However, about 10% of participants suggested changes to the Fula translation of the tool. The dialect (Fula Puta) used in the tool was not generally acceptable to the participants. Fula Torah was suggested as the appropriate dialect.

The colour, pictures and voices used in the DICCQ were acceptable to the participants (Table 3). About 60% reported it was easy to follow and 70% were comfortable with it. About 17% suggested changes to the tool mainly on reducing administration time (8%) and waiting time (9%).

Table 4 shows that the mean participants’ scores were high on the question items on adverse event/risk (4.36 ± 1.21), voluntary participation (2.86 ± 0.65), meaning of giving consent (2.93 ± 0.46), study procedures (3.33 ± 0.95) while lowest mean scores were recorded on the two question items about randomisation (0.02 ± 0.15) and (0.88 ± 1.13).

The differences in the mean scores for participants’ recall between first and second visits were statistically significant [F (1,41)=25.38, p<0.00001] (Table 5). Similarly, the mean scores for participants’ understanding between first and second visits were statistically significant [F (1,41)=31.61, p<0.00001]. Pair-wise comparison of the significance levels for the time difference of the participants’ recall scores at the two study visits showed a mean time difference of 2.33, standard error of 0.463 and p<0.0001, 95% CI (1.398-3.269). The participants’ understanding scores showed a mean time difference of 3.60, standard error of 0.639 and p<0.0001, 95% CI (2.304-4.887).

## Findings of FGDs

### Acceptability

Overall, there is a consensus that the multimedia tool was clear, helpful, informative, easy to follow and understand. Most of the participants were excited about watching the video and hearing their local languages being used to explain the study information. One male participant expressed that the tool was capable of improving understanding of study information as follows: ‘*I have been coming to this hospital for over 10 years; I have never seen a thing like this. The sound is very good and clear to me, I am sure this thing will help to improve understanding. I am happy (and) like to join (PRINOGAM) study*’.

A female participant commented: ‘*Though I have taken part in MRC studies before, but this one will be different. The picture and the information are clear, I am very impressed. My concern is if I get pregnant before the time this study starts, how will I take part?*’

### Ease of use

The majority of the participants admitted that they could not used a computer, but could use mobile phones on daily basis, which they claimed made the multimedia and DICCQ tools easy to follow and use. One of the participants noted: ‘*I must thank you people for thinking of this very nice thing. Although, I am not used to a computer, I can use mobile phones very well. (So), I can follow and even use this computer easily*’.

### Suggested changes to multimedia and DICCQ

One participant said: ‘*The video is fine but it will be better if background music is reduced*’. A male participant suggested reducing the time to administer the DICCQ and reduction in overall waiting time. ‘*I am happy with this tool,*’ he said, ‘*but you have to do something about the time (administration and waiting time), so that we can return quickly to our places of work*’.

## Discussion

This study evaluated a multimedia tool developed to obtain informed consent from low literacy participants who were potentially eligible to enrol in a clinical trial. Despite the fact that only 10% of the study population had formal education, the computerised tool was well received and easy to administer. Similarly, a digitised audio comprehension questionnaire developed in a previous study [31] was also acceptable to these participants. The participants expressed satisfaction with the tools and wanted future studies to adopt them. However, they suggested reducing the administration time for the digitised questionnaire, overall waiting time and background music in the multimedia.

The mean participants’ scores were relatively high on the question items about adverse events/risk, voluntary participation, meaning of giving consent and study procedures, implying that the participants understood these elements better. Conversely, the scores were lowest on the items on randomisation showing that the participants had least understanding on this domain. Illustrations of the study information using a combination of video, animations and oral explanation in local languages could have contributed to the high comprehension scores recorded by participants in this study. This finding represents a new insight into the use of multimedia tool to deliver consent information to low literacy participants in sub-Saharan Africa.

Furthermore, the multimedia tool increased significantly both recall and understanding scores of the participants and this is consistent with the results from some previous studies [34-38]. The increase in participants’ recall and understanding scores observed after one week period could be explained by the quiz/feedback strategy adopted in the digitised questionnaire. This introduced the possibility of enhancement or practice effect due to memorisation which might occur when participants gave correct answers or when the researchers clarified area of concerns. To minimise the memorisation or practice effect, the digitised questionnaire used closed ended, multiple-choice and open-ended items which were likely to elicit responses that truly reflect participants’ comprehension of the information.

A major benefit of the multimedia tool is that it consistently provides the same research information to all participants in the same manner. This strategy removes inter-person variations in translations of informed consent information to the low literacy research participants. This becomes crucial as a participant’s comprehension is influenced by the communication skills of the person administering the consent. This is truer in contexts like the Gambia, where there is no standard writing format for the local languages and the person administering the consent plays a key role in translating it orally. It was therefore critical that we employed the services of experienced linguistic professionals who were native speakers to translate the written English version of the informed consent document to the audio forms of three major Gambian languages.

Furthermore, the development of the multimedia tool involved many technical processes including graphical translation of elements of the informed consent document to appropriate visual stories. These were further acted in role-plays by trained individuals before video, animations and audio-translations in local languages are systematically added. Some researchers have argued that the time and cost involved in the production of a multimedia tool might further add to logistic challenges of the conduct of clinical trials [39,40]. However, the ultimate benefits of ensuring well-informed research participants through the use of multimedia intervention could, in addition to improving participants’ comprehension, protect their freedom to decide, and also potentially improve the quality of data and outcome of the research. This remains a worthy venture even if it requires spending a little more than expected.

The use of a multimedia tool to deliver study information during informed consent process may weaken compassionate human interactions that form the basis of research ethics [41]. Therefore, it could be counter-productive to depend solely on the technology to meet the information needs of participants during the informed consent process. The research team need to keep enough time in discussing the participants’ concerns about the research, in addition to the multimedia. The multimedia could in fact replace the first part of ‘traditional’ consent interview. It could be followed by an interview where the participants would still be free to ask clarification questions. Thus, the overall acceptance and success of the tool will ultimately depend on a well-balanced combination of the technology and human elements.

Although both quantitative and qualitative assessments adopted in our study consistently revealed improvements in participants’ comprehension scores, caution is required in interpreting these observations because our study targeted healthy volunteers who were not enrolled in any study. The simulated trial situation might have over-estimated the advantages of the multimedia tool and under-estimated other factors which would be present in real life, e.g. the participants’ anxiety and haste to get enrolled. Nevertheless, increases in participants’ comprehension scores over a one-week period were consistent with the design of this study. The use of repeated measures design allowed the study participants to serve as their own control. This improves the precision of the study by reducing the size of the error variance.

Another limitation of our study may be due to the fact that it reflects the situation in The Gambia, where local languages do not have written standardised forms. Consequently, we suggest that the tool should be later tested and adapted in different sub-Saharan African contexts.

Our study provides important information on the development and evaluation of a multimedia strategy for improving comprehension in research informed consent to low literacy individuals. Information shared on processes involved in the development will serve as a useful guide for investigators in similar settings. Another study comparing the multimedia consent to the ‘conventional’ consent procedure among participants enrolled for the parent trial is currently ongoing. We hope findings of the study will shed more lights on the efficacy of multimedia in a real-life setting.

## Conclusions

This study developed and evaluated a multimedia informed consent tool for improving the informed consent procedure, and noteworthy comprehension, in low literacy participants in The Gambia. We carried out an initial assessment of the strength of the tool and identified areas for further research and improvement. This study represents an important step towards institutionalising a context-specific informed consent tool. Future work is needed but there was enthusiasm for this modality and potential to improve the informed consent process, leading in turn to a better and more solid partnership between the community and the clinical researchers in The Gambia.

## Figures and Tables

**Table 1:** Socio-demographic characteristics of the study participants.

Characteristics	Frequency (%) N=42

**Age group**	
18-25 years	4(9.5)
26-33 years	16(38.1)
34-41 years	13(31.0)
42-49 years	9(21.4)

**Sex**	
Female	20(47.6)
Male	22(52.4)

**Ethnicity**	
Mandinka	38(90.5)
Fula	4(9.5)

**Highest level of education attained**	
Primary	2(4.8)
Secondary	2(4.8)
Arabic	29(69.0)
Vocational education	1(2.4)
No formal education	8(19.0)

**Occupation**	
Artisan	7(16.7)
Farming	10 (23.8)
Housewife	17(40.5)
Schooling	1 (2.4)
Trading	7(16.7)

**Area of domicile**	
Basse North	20(47.6)
Basse South	22(52.4)

**Table 2:** Participants’ responses to questions on acceptability and ease of use of multimedia informed consent tool.

**1. Overall, how much do you like the following features of the multimedia tool?**	**Like (N=42)**	**Dislike (N=42)**	**I don’t know (N=42)**
Colour	42(100.0)	0(0.0)	0(0.0)
Pictures	42(100.0)	0(0.0)	0(0.0)
Voices	42(100.0)	0(0.0)	0(0.0)
Duration	42(100.0)	0(0.0)	0(0.0)
**2. Do you think the tool provide enough information about the study?**	**Yes**	**No**	**I don’t know**
	42(100.0)	0(0.0)	0(0.0)
**3. Overall, how comfortable are you with the information in the tool?**	**Comfortable**	**Very comfortable**	**Not comfortable**
	30(71.4)	12(28.6)	0(0.0)
**4. Overall, how easy or difficult did you find the Information provided In the tool**	**Easy**	**Very easy**	**Difficult**
	30(71.4)	12(28.6)	0(0.0)
**5. Will you like to use it again?**	**Yes**	**No**	**I don’t know**
	42(100.0)	0(0.0)	0(0.0)
**6. Would you want future study information delivered through this tool?**	**Yes**	**No**	**I don’t know**
	42(100.0)	0(0.0)	0(0.0)
**7. Do you want any changes to the tool?**	**Yes**	**No**	**I don’t know**
	4(9.5)	38(90.5)	0(0.0)

Table 2 shows that all participants liked the features of the multimedia tool, about 70% found it easy to follow and 10% suggested some changes to the tool

**Table 3:** Participants’ responses to questions on acceptability and ease of use of digitised comprehension questionnaire (DICCQ).

**1. Overall, how much do you like the following features of DICCQ?**	**Like (N=42)**	**Dislike (N=42)**	**I don’t know (N=42)**
Colour	42(100.0)	0(0.0)	0(0.0)
Pictures	42(100.0)	0(0.0)	0(0.0)
Voices	42(100.0)	0(0.0)	0(0.0)
Duration	39(92.9)	3(7.1)	0(0.0)
**2. Overall, how easy or difficult did you find the questions provided In the tool**	**Easy**	**Very easy**	**Difficult**
	24(57.1)	18(42.9)	0(0.0)
**3. Overall, how comfortable are you with the information in the tool?**	**Comfortable**	**Very comfortable**	**Not comfortable**
	30(71.4)	11(26.2)	1(2.4)
**4. Will you like to use it again?**	**Yes**	**No**	**I don’t know**
	42(100.0)	0(0.0)	0(0.0)
**5. Would you want future study questionnaires delivered through this tool?**	**Yes**	**No**	**I don’t know**
	42(100.0)	0(0.0)	0(0.0)
**6. Do you want any changes to the tool?**	**Yes**	**No**	**I don’t know**
	7(16.7)	35(83.3)	0(0.0)

Table 3 shows that majority of the participants liked the features of the audio questionnaire, about 60% found it easy to follow and 17% suggested changes to the tool

**Table 4:** Descriptive statistics of participants’ scores on the audio digitised comprehension questionnaire (DICCQ).

**Section A: Choose only one right answer**		**Mean**	SD*	**Minimum**	**Maximum**
**1.**	Have you been told that you can freely decide to take part in this study?	2.86	0.65	0	3
**2**	Have you been told you can withdraw from this study anytime?	2.74	0.83	0	3
**3.**	During the study, will you know the drug you or your child is receiving?	2.86	0.52	1	3
**4.**	If yes, describe or mention what the drug is doing?	2.95	1.10	1	5
**5.**	During the study, will anyone not working with MRC know about your health information?	2.29	1.23	0	3
**6.**	Have you been given the name and phone number of the person to contact if you have any questions about the study?	2.50	0.99	0	3
**7.**	If yes, mention the name of the person?	2.07	1.30	0	3
**8.**	Can your participation in the study be stopped without your consent?	1.49	1.49	0	3
**9.**	Will you receive money for taking part in the study?	2.52	0.94	0	3
**Section B: Answer the following questions by circling the right answer**					
**10.**	How were participants divided into different groups in this study?	0.02	0.15	0	1
**11.**	At what point can you leave the study?	2.02	1.41	0	3
**12.**	What does it mean when you sign or thumbprint the study consent form?	2.93	0.46	0	3
**13.**	How did you decide to participate in this study?	1.79	1.49	0	3
**14.**	What will you receive as a reward for taking part in the study?	2.79	0.78	0	3
**15.**	What will happen if you decide to stop taking part in this study?	2.71	0.89	0	3
**Section C: You will need to circle more than one correct answers in this part**					
**16.**	Which of the following describes why the primaquine study is being done?	2.95	1.01	2	4
**17.**	Which procedures were you asked to take part in?	3.33	0.95	2	4
**18.**	Which activities were you asked to complete?	2.76	0.98	2	4
**19.**	Which describes the main benefits of taking part in the study?	3.04	1.01	2	4
**Section D: In this section, you are requested to provide answers that are specific to the study you are currently participating**.					
**20.**	Please tell me what the researchers want to find out in the study?	2.92	0.78	0	5
**21.**	How many times do you have to come to the clinic for a visit during the study?	3.17	2.17	0	5
**22.**	Tell me what will be done during the study visits?	2.92	1.26	0	5
**23.**	How are participant assigned into different groups this study?	0.88	1.13	0	3
**24.**	What is the difference between taking part in this study and going to see a doctor for treatment?	2.97	2.48	0	3
**25.**	What are the possible unwanted effects of taking part in this study?	4.36	1.21	0	5
**26.**	Why do you think some of the study participants were given different medicine?	2.98	2.48	0	3

**Table 5:** Repeated measures analysis of variance of participants’ recall and understanding scores.

	‘Recall’ scores (N=42)	‘Understanding’ scores (N=42)
**1^st^ visit**	25.62±4.4	55.00±5.58
**2^nd^ visit**	27.95±4.8	58.5952±7.06
**Within-participant effects**	F-test=25.38 P<0.001	F-test=31.61 P<0.001
**Between-participant effects**	F-test=1588.91 P<0.0001	F-test=3743.267 P<0.0001
**Pairwise comparison of significance levels for the time difference**	Mean time difference =2.33 S.E=0.463, P<0.0001 95% CI (1.398-3.269)	Mean time difference= 3.60 S.E=0.639, P<0.0001 95% CI (2.304-4.887)
